# *In Silico* Analysis of the Apolipoprotein E and the Amyloid *β* Peptide Interaction: Misfolding Induced by Frustration of the Salt Bridge Network

**DOI:** 10.1371/journal.pcbi.1000663

**Published:** 2010-02-05

**Authors:** Jinghui Luo, Jean-Didier Maréchal, Sebastian Wärmländer, Astrid Gräslund, Alex Perálvarez-Marín

**Affiliations:** 1 Department of Biochemistry and Biophysics, Stockholm University, Stockholm, Sweden; 2 Unitat de Química Física, Departament de Química, Universitat Autònoma de Barcelona, Bellaterra, Spain; 3 Unitat de Biofísica, Departament de Bioquímica i de Biologia Molecular i Centre d'Estudis en Biofísica, Universitat Autònoma de Barcelona, Bellaterra, Spain; Harvard University, United States of America

## Abstract

The relationship between Apolipoprotein E (ApoE) and the aggregation processes of the amyloid *β* (A*β*) peptide has been shown to be crucial for Alzheimer's disease (AD). The presence of the ApoE4 isoform is considered to be a contributing risk factor for AD. However, the detailed molecular properties of ApoE4 interacting with the A*β* peptide are unknown, although various mechanisms have been proposed to explain the physiological and pathological role of this relationship. Here, computer simulations have been used to investigate the process of A*β* interaction with the N-terminal domain of the human ApoE isoforms (ApoE2, ApoE3 and ApoE4). Molecular docking combined with molecular dynamics simulations have been undertaken to determine the A*β* peptide binding sites and the relative stability of binding to each of the ApoE isoforms. Our results show that from the several ApoE isoforms investigated, only ApoE4 presents a misfolded intermediate when bound to A*β*. Moreover, the initial α-helix used as the A*β* peptide model structure also becomes unstructured due to the interaction with ApoE4. These structural changes appear to be related to a rearrangement of the salt bridge network in ApoE4, for which we propose a model. It seems plausible that ApoE4 in its partially unfolded state is incapable of performing the clearance of A*β*, thereby promoting amyloid forming processes. Hence, the proposed model can be used to identify potential drug binding sites in the ApoE4-A*β* complex, where the interaction between the two molecules can be inhibited.

## Author Summary

Unraveling the molecular details of the interaction between apolipoprotein E and the amyloid β peptide will yield insights into the relationship between Alzheimer's disease and lipid transport and metabolism. The isoform E4 of apolipoprotein E has been shown to be closely related to Alzheimer's disease. We have therefore used a computational approach to depict a detailed interaction map for this peptide-lipoprotein interaction. The simulation shows that the specific formation of the lipoprotein isoform E4 and the peptide complex affects the structure of the lipoprotein and the peptide. We suggest that this is related to some of the pathogenic effects in Alzheimer's disease. Our results provide a molecular model to work with for the design of potential therapeutic agents capable of modulating this interaction.

## Introduction

Alzheimer's disease (AD) is one of the most common neurodegenerative diseases at the present time. The disease is characterized by the formation of neurofibrillary tangles and plaques in the brain, leading to neuronal dysfunction, neuronal loss and finally death. The main component of the plaques is the amyloid-*β* peptide (A*β*), a 39–43 amino acids long hydrophobic peptide generated by the cleavage of the amyloid precursor, which accumulates in the form of soluble and non-soluble aggregates.

The connection between Apolipoprotein E (ApoE) and AD is well established [1],[2]. Structurally, ApoE is a 299 residues protein with an N-terminal domain involved in binding to heparin, low density lipoprotein receptors (LDLR) and LDLR-related proteins [3],[4]. The C-terminal domain has been related to heparin and lipid binding [5],[6]. Three main isoforms have been described for human ApoE, i.e. ApoE2, ApoE3 and ApoE4. The standard variant is ApoE3, while ApoE2 is defective for receptor binding, causing *APOE* ε2/ε2 homozygotic individuals to have a higher predisposition to diseases related to high amounts of cholesterol and triglycerides [3],[7]. For ApoE4, the receptor binding affinity remains unaffected, but *APOE* ε4/ε4 homozygotic individuals have higher risk for coronary heart disease and a significantly greater risk for developing AD.[1],[8] Around 80% of all AD cases are related to the genetic variance at the ApoE locus [9],[10].

**Figure 1:**
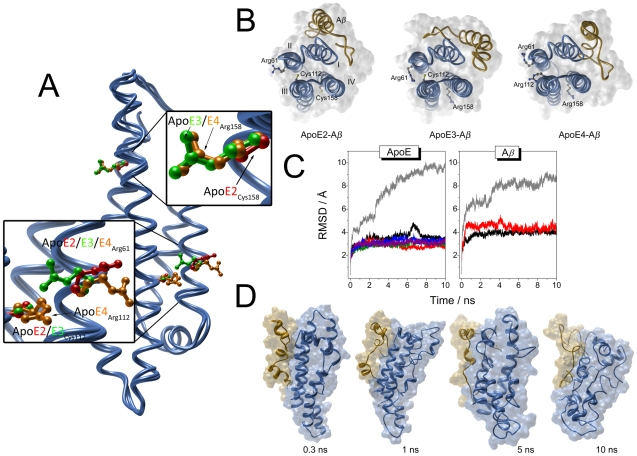
Computational docking and molecular dynamics for ApoE and A*β*. (A) Comparison of the crystal structures of the N-terminal domain of the different ApoE truncated isoforms. The α-carbons of the different ApoE isoform crystal structures were aligned and plotted as ribbons. Residues 112 and 158, which variability leads to the different isoforms have been plotted as ball and sticks. For a clearer representation, the most representative areas of the protein have been scaled in the insets. The atoms of the corresponding side chains have been colored using the following color code: red for ApoE2, green for ApoE3 and orange for ApoE4. Arg61 has been also plotted using the same representation mode and color code. (B) Docking of Apolipoprotein E with A*β* peptide. Isoforms E2, E3 and E4 models with lowest global energy docked with the A*β* peptide are represented. The surface corresponding to the occupancy of both ApoE and A*β* is represented in white. ApoE and A*β* are represented by blue and golden cartoons, respectively. Residues 61, 112 and 158 have been represented as ball and sticks and colored by element (C, grey; S, yellow; N, blue). (C) RMSDs for ApoE and A*β* peptide by molecular dynamics with GROMACS. (ApoE) RMSD values of the dynamics for ApoE complexed or not with A*β* peptide. ApoE alone: ApoE2, black; ApoE3, red; ApoE4, green. ApoE-A*β* complex: ApoE2- A*β*, blue; ApoE3- A*β*, purple; ApoE4-A*β*, grey. (A*β*) RMSD values of the dynamics for the A*β* peptide complexed with ApoE: ApoE2- A*β*, black; ApoE3- A*β*, red; ApoE4- A*β*, grey. (D) Snapshots from the ApoE4-A*β* complex formation during the MD simulation. Plot of the folding intermediates generated by the MD simulation at the indicated times. ApoE and A*β* are represented by blue and golden surfaces/cartoons, respectively. It is noteworthy the loss of secondary structure as a function of time.

The only difference between the ApoE isoforms is found in residues 112 and 158, where Cys112/Cys158 corresponds to ApoE2, Cys112/Arg158 to ApoE3, and Arg112/Arg158 to ApoE4. The presence of cysteines at these positions confers oligomerization properties to ApoE. Indeed, ApoE2 and ApoE3 are able to form disulfide-linked homo- and hetero-oligomers due to the presence of “respectively” two and one Cys residue. ApoE4 lacks the possibility of strong disulfide linking; however, it is unclear whether weaker interactions could promote the oligomerization of ApoE4. The Cys/Arg substitution in ApoE4 also has molecular impact in terms of intra-protein polar contacts: the orientation of Arg61 is different in ApoE4 compared to ApoE3; the orientation of Arg61 towards the C-terminal domain (See Figure 1A) facilitates a salt bridge between Arg61 and Glu255. The electrostatic interaction between Arg61 and Glu255 promotes an N- and C-domain interaction that packs the structure tighter, which seems crucial for the interaction of ApoE4 with triglyceride-rich lipoproteins. The interaction between Arg61 and Glu255 is absent in ApoE3 leading to a more open structure and a preferential binding of phospholipid-rich high-density lipoproteins [11],[12]. Chemical and thermal denaturation experiments have shown that the most unstable structure belongs to ApoE4, which displays a partially unfolded intermediate (molten globule) containing some β structure that may be related to the fact that ApoE4 enhances the deposition of A*β* [13],[14].

Although different mechanisms have been proposed to explain the physiological and pathological relationship between ApoE and the A*β* peptide, the details of the interaction between ApoE and A*β* at a molecular level are unknown. Such detailed knowledge is however important for the understanding of the pathological mechanisms of AD, and may also help to identify potential therapeutic target sites where the interaction between ApoE4 and A*β* can be blocked.

In the present study we are using molecular docking simulations based on global minimum energy to investigate the interaction process of A*β* with the N-terminal domain of the different ApoE isoforms in order to determine potential A*β* peptide binding sites in ApoE. In the next step, molecular dynamics (MD) calculations are undertaken to explore the conformational dynamics of ApoE under A*β* interaction and evaluate the stability of each of the ApoE-A*β* complexes. From the analysis and the statistics of the electrostatic interactions of the three ApoE isoforms, we present a model explaining the role of the A*β*-ApoE interaction and its relevance for AD.

## Results

Molecular dockings followed by MD simulations were used to study the interaction of A*β* with the different isoforms of ApoE. In order to study the A*β* peptide binding site on the N-terminal domains of the three ApoE truncated isoforms we used the A*β*(1–40) peptide as ligand, employing an SDS-induced α-helix solution structure previously determined by NMR spectroscopy [15]. Indeed, such helical fold in the A*β* monomeric state (non-aggregated) has been shown to be the most populated one in highly hydrophobic environments [16]. On the other hand, the structures of the three ApoE truncated isoforms were taken from lipid-free structure determinations by X-ray crystallography [11],[17],[18], which correspond only to the N-terminal domain (144 residues including the LDLR domanin of ApoE). Water molecules in the pdb files were removed prior to docking and energy minimizations were carried out to refine the structures.

All 3D models of the ApoE-A*β* complexes were found to be quite different. Although the A*β*(1–40) peptide assembles between the first and fourth ApoE helix for all ApoE isoforms, the orientation of the peptide was found to depend on the ApoE variant (Figure 1B; see Figure S1 for comparison of the 10 lowest energy solutions for each isoform). For ApoE2 and ApoE4, the C-terminus of the peptide faces the N-terminus of the protein, though the assembly is different. For ApoE3, the peptide is turned around, and the N-terminus of the peptide faces the N-terminus of the protein. Early studies indicated that ApoE interaction with A*β* fibrils is partially dependent on ionic interactions [19]. Thus, the single change of Cys158 in ApoE2 to Arg158 in ApoE3 changes the distribution of ionic residues influencing the assembly of A*β* (1–40), while the double change of Cys112 and Cys158 to Arg112 and Arg158 in ApoE4 distributes the ionic residues in an ApoE2-like way.

A 10 ns classical MD simulation including explicit water of the three ApoE isoforms together with the A*β* peptide was carried out on each of the lowest energy ApoE-A*β* models obtained by docking calculations as well as on each isolated species. Figure 1C shows the root-mean-square deviation (RMSD) of the MD simulation for the three ApoE isoforms in the presence and absence of the peptide. In their unbound form, no conformational transitions were detected for the ApoE isoforms, in agreement with previous results [20]. However, in presence of the peptide, different behaviors were observed between the isoforms. Despite the existence of interaction, no conformational transitions were detected for the ApoE2-A*β* or the ApoE3-A*β* complexes. However, the ApoE4-A*β* complex showed a large conformational transition indicated by a significant RMSD change of about 10 Å in the 10 ns timescale (Figure 1C).

In Figure 1D, four snapshots of the 10 ns MD simulation for the ApoE4-A*β* complex are presented. Focusing on ApoE4, during the first 0.3 ns, the third helix of ApoE4 started to unfold and a loop appeared between residues 112 and 92 which affected the whole third helix. This structural disturbance was caused by the onset of new electrostatic interactions rising from the interaction with the peptide. For the A*β* peptide, the first conformational change appeared in the Glu22-Asp23 region. At 1ns the second helix of ApoE4 showed a conformational change. In the snapshots of 5 ns, the first and fourth helices of ApoE4 were still stable, but at 10 ns a large conformational change had occurred, coinciding with a fully extended A*β*(1–40) peptide. At 10 ns, the hydrophobic groups inside the ApoE4 helices had become exposed to the solvent. The interruption of the stable salt bridge network by external electrostatic interactions (coming from the peptide) was thus transmitted from the dense helix region to the whole protein, causing a severe loss of α-helical structure.

**Figure 2:**
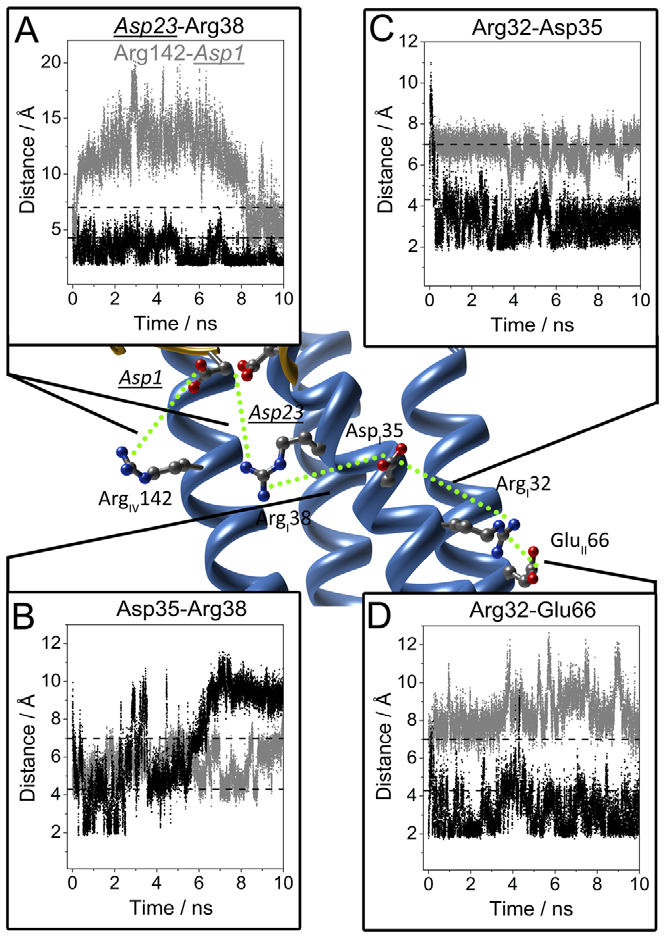
Distance analysis between *Aβ* and ApoE residues involved in the electrostatic interactions during the ApoE-A*β* complex formation. The structure plotted corresponds to the ApoE4-A*β* complex (color code blue and golden, respectively). A*β* residues are indicated by underlined and italics characters. A*β* peptide residues from Gly25 to Val40 have been removed for a clearer representation. The sub index for the ApoE4 residues indicates helix location. The green dotted line depicts the salt bridge network between residues of the A*β* peptide and ApoE4 and between residues in the ApoE4 helices I and II. (A) Distance variation during the 10 ns MD simulation for the A*β* Asp23 and the ApoE4 Arg38 electrostatic pair (black) and the A*β* Asp1 and the ApoE4 Arg142 electrostatic pair (grey). (B) Distance variation during the 10 ns MD simulation for the Asp35-Arg38 electrostatic pair, for ApoE4 alone (grey) and for ApoE4-A*β* (black). (C) Distance variation during the 10 ns MD simulation for the Arg32-Asp35 electrostatic pair, for ApoE4 alone (grey) and for ApoE4-A*β* (black). (D) Distance variation during the 10 ns MD simulation for the Arg32-Glu66 electrostatic pair, for ApoE4 alone (grey) and for ApoE4-A*β* (black). In all plots the salt bridge thresholds of 4.3 and 7.0 Å are indicated by dashed lines. Selected residues have been represented as ball and sticks and colored by element (C, grey; O, red; N, blue).

Further investigation on the conformational change induced in ApoE4 by the complexation with A*β* was carried out through the analysis of the distances between charged residues. For this analysis, direct salt bridges have been assumed to be around 4.3 Å, whereas indirect or water-mediated salt bridges have been assumed to have a distance between 4.3 and 7.0 Å as reported by Dzubiella *et al.* [21]. In the most stable ApoE4-A*β* complex, the peptide interacted with helices I and IV of ApoE4. The A*β* residues responsible for these interactions were the negatively charged Asp1 and Asp23, which interacted with positively charged arginines in ApoE4 (Arg38 in helix I and Arg142 in helix IV respectively). The direct salt bridge between A*β*_Asp23_ and ApoE4_Arg38_ was very strong (Figure 2A), while the salt bridge between A*β*_Asp1_ and ApoE4_Arg142_ did not exist during most of the MD simulation, and only became more plausible at the end of the MD simulation (the distance for an indirect salt bridge being reached after circa 8 ns, Figure 2A). Focusing on helices I and II of the N-terminal domain of ApoE4, the distance between Arg38 and Asp35 changed during the 10 ns time window (see Figure 2B). A transition occurred from 10 to 2.5 Å in the 2 ns time window, which then went back to 10 Å (indicating the breaking of the Arg38-Asp35 salt bridge), and became stable at 7 ns. For comparison, the same distance is shown for the MD simulation of ApoE4 alone, where no change at all can be seen, as the distance was within the 4.3 and 7.0 Å range during the whole 10 ns (Figure 2B). The salt bridge between Asp35 and Arg32 was stable below 4.4 Å before 2 ns (Figure 2C). For ApoE4 in the absence of A*β*, the distance remained constant around the 7.0 Å threshold, making it difficult to determine the existence of an indirect salt bridge. For the ApoE4-A*β* complex, the direct salt bridge involving Arg32 and Glu66 (in helices I and II, respectively) was affected and showed a maximal fluctuation from 2.5 to 7.5 Å and then back to 2.5 Å in the 10 ns time window (Figure 2D). In the ApoE4 alone MD, this Arg32-Glu66 pair did not show any propensity to interact (the distance was over 7.0 Å during the whole 10 ns).

**Figure 3:**
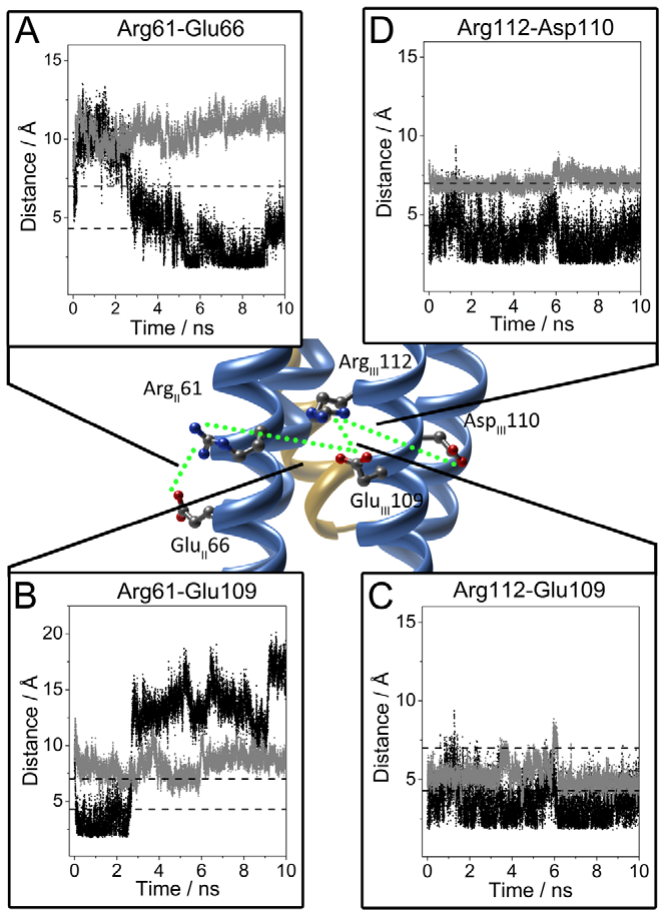
Distance analysis between ApoE residues involved in the electrostatic interactions between helices II and III of the N-terminal domain during the ApoE-A*β* complex formation. The structure plotted corresponds to the ApoE4-A*β* complex (color code blue and golden, respectively). The sub index for the ApoE4 residues indicates helix location. The green dotted line depicts the salt bridge network between residues in the ApoE4 helices II and III. (A) Distance variation during the 10 ns MD simulation for the Arg61-Glu66 electrostatic pair, for ApoE4 alone (grey) and for ApoE4-A*β* (black). (B) Distance variation during the 10 ns MD simulation for the Arg61-Glu109 electrostatic pair, for ApoE4 alone (grey) and for ApoE4-A*β* (black). (C) Distance variation during the 10 ns MD simulation for the Arg112-Glu109 electrostatic pair, for ApoE4 alone (grey) and for ApoE4-A*β* (black). (D) Distance variation during the 10 ns MD simulation for the Arg112-Asp110 electrostatic pair, for ApoE4 alone (grey) and for ApoE4-A*β* (black). In all plots the salt bridge thresholds of 4.3 and 7.0 Å are indicated by dashed lines. Selected residues have been represented as ball and sticks and colored by element (C, grey; O, red; N, blue).

For helices II and III of the N-terminal domain of ApoE4, the transitions of the Arg61-Glu66, Arg61-Glu109 and Glu109-Arg112 salt bridges were monitored in the ApoE4-A*β* complex (see Figure 3). At 5 ns the distance between Glu66 and Arg61 from helix II dropped from about 10 to 3 Å, becoming stable and forming a direct salt bridge (see Figure 3A). However, for ApoE4 alone, this salt bridge was never formed. For the ApoE4-A*β* complex in the 5 ns interval, the direct salt bridge between Arg61 and Glu109 (helix III) broke down (the distance increased from about 3 to 12.5 Å, Figure 3B). In ApoE4 alone the distance for this pair was out of range during most of the MD simulation. However, the distance between Glu109 and Arg112 (both in helix III) remained relatively stable and below the salt bridge distance threshold (Figure 3C). In the ApoE4-A*β* complex, the Glu109-Arg112 salt bridge was direct (below 4.3 Å), whereas for ApoE4 alone, the salt bridge was more indirect or water mediated. The MD results for the Arg112-Asp110 pair (Figure 3D) were similar to those for the Arg112-Glu109 pair. In the complex, the distance for the electrostatic pair indicated a direct salt bridge, whereas for ApoE4 alone, this distance was closer to an indirect salt bridge (if any).

**Figure 4:**
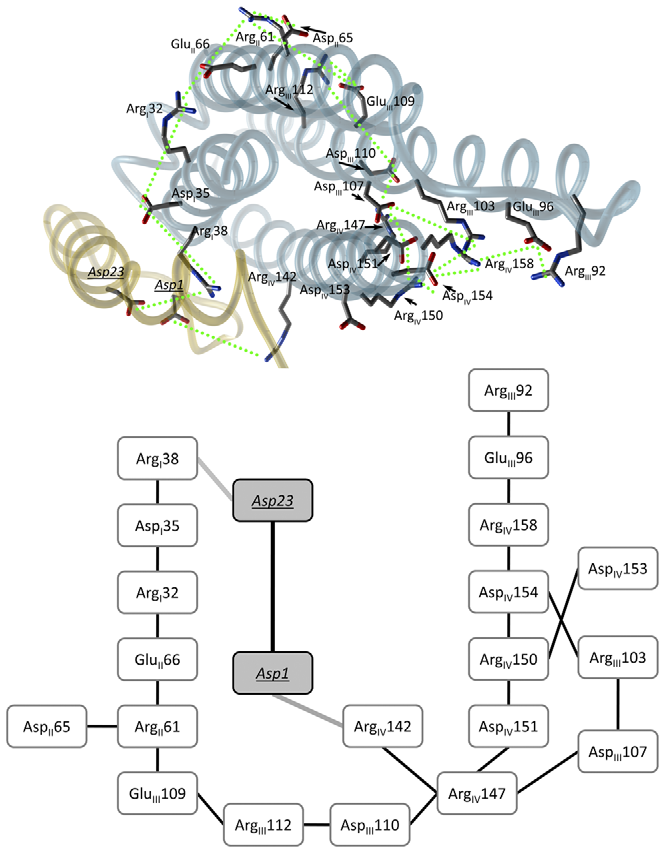
Proposed model for the A*β* peptide and ApoE4 interaction. The interaction of A*β* with ApoE4 rearranges the salt bridge network. This is the overall representation of the interaction effect between the peptide and the protein. (*Upper panel*) 3D plot of the ApoE4-A*β* complex (colored blue and golden respectively) depicting the rearranged salt bridge network (green dotted lines). Selected residues have been represented as ball and sticks and colored by element (C, grey; O, red; N, blue). (*Lower panel*) 2D scheme for a clearer understanding of the rearranged salt bridge network. The sub index for the ApoE4 residues indicates helix location. A*β* residues into grey shaded boxes are indicated by underlined and italics characters.

Electrostatic interactions between helix III and helix IV were more complex and insensitive to the interaction with the peptide, and the bridge network involving helices III and IV remained stable during the simulation (data not shown). In the ApoE4-A*β* complex, the interaction between Arg112 with Asp110 and Glu109 in helix III is connected to helix IV via the Asp110-Arg147 and Asp107-Arg151 ion pairs (see Figure 4). Also Asp107 in helix III and Asp151 in helix IV interacted with Arg147. Another inter-helical ion pair network existed between Arg103, Glu96 and Arg92 in helix III and Arg150, Arg153, Arg154 and Arg158 in helix IV (see Figure 4). Arg158 acted as a bridge for extending the electrostatic interaction between Glu96 and Arg92.

## Discussion

### The model of electrostatic interaction between A*β* peptide and ApoE4

Our computational approach assumes a direct interaction between ApoE and A*β*. Although the docking was plausible for ApoE2 and ApoE3, the interactions did not generate any conformational transition in the 10 ns time window while for ApoE4, the interaction promoted unfolding of the ApoE4, as shown by the MD simulations. This result is compatible with earlier thermal and chemical denaturation studies using circular dichroism and scanning calorimetry, which have indicated stability differences (ApoE4<ApoE3<ApoE2) among the three isofoms (experiments were carried out on the 22 KDa truncated protein, corresponding to the N-terminal domain) [13],[14]. The present results also agree with the existence of a partially unfolded intermediate for ApoE4 [22]. However, a direct comparison of the present results with the previous experimental results is not possible. The MD results for the ApoE isoforms alone do not indicate any of the trends shown experimentally, probably because of the time scale (nanoseconds *vs.* seconds/minutes). But in the case of ApoE4, it is likely the A*β* peptide behaves as an unfolding catalyzer. Thus, effects on the stability of ApoE2 and ApoE3 exerted by A*β* peptide at longer time scale cannot be discarded.

The proposed ApoE4-A*β* complex forms between helices I and IV of ApoE4 (proposed model in Figures 1B and 4). As seen from the docking procedure, the complex formation does not directly affect the salt bridges involving Arg61, but the cascade of events generated by the interaction leads to the stabilization and destabilization of the Arg61-Glu66 and Arg61-Glu109 salt bridges, respectively. Arg112 in ApoE4 causes the side chain of Arg61 to extend away from the four–helix bundle which will allow electrostatic interaction with Asp65, Glu66 and Glu59 (see Figure 4). In ApoE2 and ApoE3, Arg61 shows a different orientation (due to Cys122, see Figure 1A), hindering the interaction with the charged residues from helix III.

The fluctuation of the salt bridges in helices I and II could be explained by the interruption of the Arg38-Asp25 salt bridge in ApoE4. This effect is most likely induced by Asp23 of A*β*, which will affect the neighboring salt bridge between Asp35 and Arg32. Another affected interaction would be the inter-helix salt bridge between Arg32 (helix I) and Glu66 (helix II). The MD simulations show that this initial chain of events induced by the presence of the A*β* peptide and occurring in helices I and II of ApoE4 (but not ApoE3) would soon be transmitted to helix III stabilizing the Arg61-Glu66 and breaking the Arg61-Glu109 salt bridges in this N-terminal domain, and probably affecting also the Arg61-Glu255 salt bridge in the full protein form. Disruption of this domain interaction by the ApoE4 R61T mutation has been shown to reduce A*β* production [23]. In the same study, an ApoE4 docking site involving residues 109, 112 and 61, was defined as a binding site for blocking agents capable to disrupt the domain interaction leading to a decrease in A*β* production [23]. The other contact point comprising A*β*_Asp1_ and ApoE4_Arg142_ appears less relevant for the destabilization of the salt bridge network; however, Arg142 is within the heparin and receptor binding region (localized around residues 141–150 of ApoE). This direct interaction may shield the ApoE4 binding region, affecting the cell membrane recognition of ApoE4 interacting with A*β*.

As shown by *in vitro* studies, both ApoE3 and ApoE4 interact with A*β* and form SDS stable complexes. ApoE-A*β* complexes have been isolated from AD brain extracts and shown to be stable and as tightly packed as A*β* fibrils [24],[25]. Our results indicate the possibility that both ApoE3 and ApoE4 bind to the peptide with different orientations. Assuming the protective role of ApoE3 compared to the detrimental role of ApoE4 in AD (for an extensive review see Huang et al. [26]), we can speculate the following: the binding of the peptide with ApoE3 does not affect the stability of the protein nor the complex, leading to the peptide clearance. On the other hand, the lower stability of ApoE4 is even more emphasized by the interaction with A*β*: the interaction triggers the partial unfolding of ApoE4 into a misfolded intermediate which we suggest is incapable of performing the clearance of A*β*, and leading to pathogenic effects such as the promotion of amyloid forming processes. In our results, mostly the N-terminus of the peptide is involved in the ApoE4-A*β* complex formation (residues 1 and 23). Previous studies with A*β* peptide have shown that electrostatic interactions are the main cause for the formation of larger oligomers and that the C-terminus region is important for the formation of such oligomers [27]. Discrete MD simulations have shown that the Gly37-Gly38 turn plays an important role in the formation of A*β* (1–42) pentamers [28]. Thus, we can speculate that the non-involvement of the C-terminus in the complex formation could favor the interaction of free A*β* C-termini, thus provoking the aggregation of the ApoE4-A*β* complexes.

This A*β* effect could probably be overcome by the usage of agents (such as GIND-25 and GIND-105) [23] binding to the Arg61/Glu109/Arg112 ApoE4 binding site, which would stabilize the protein by disrupting the Arg61-Glu255 salt bridge, generating an ApoE3-like variant. In the same way, A*β* and ApoE derived peptides have also been used as blocking therapeutic agents of both the protein and the peptide [29],[30].

### Conclusions

We propose that the interaction of A*β* with ApoE4 induces a partially unfolded intermediate by the frustration of the existent network of salt bridges. The four-helix bundle of ApoE4 opens up and the hydrophobic core becomes exposed due to the ApoE4-A*β* complex formation, presumably rendering the protein incapable of performing A*β* clearance. The interaction with A*β* affects the proposed binding site formed by Arg61/Glu109/Arg112 in ApoE4, a binding site that has been shown to be relevant for substances capable of reducing the A*β* production. The model here presented has implications for therapeutic drug design for AD, as it defines on a molecular level the ApoE-A*β* complex as a potential drug target.

## Methods

### Model description

Crystal structures of the three ApoE truncated isoforms (containing only the N-terminal domain) were downloaded from the PDB database (ApoE2, E3, E4, respective ID's: 1LE2, 1LPE and 1LE4), together with the A*β* peptide solution structure, determined by NMR in 10% SDS/Water (ID:1BA4) and used as the docking model. Crystallographic waters were removed and the structures were fully solvated before energy minimization. Energy minimization was performed for the macromolecules using the GROMACS3.3.2 software with GROMOS96 as the force field [31]. The RMSD between the initial and the energy minimized structures was lower than 0.01 Å for the ApoE isoforms. For the A*β* peptide, due to the flexibility of the N-terminus, the RMSD was 4.7 Å (RMSD of 0.8 Å for the α-helix A*β* residues 13 to 40).

### Docking

The structures obtained after energy minimization were used in PatchDock (http://bioinfo3d.cs.tau.ac.il/), where candidate solutions were generated by rigid-body docking methods [32],[33]. PatchDock determined the best starting candidate solutions based on shape complementarity of soft molecular surfaces. The Clustering RMSD was 4.0 Å for analysis and the complex type was set to default. The PatchDock algorithm divides the Connolly dot surface representation of the molecules into concave, convex and flat patches. Then, complementary patches are matched in order to generate candidate transformations [32],[33]. Each candidate transformation is further evaluated by a scoring function that considers both geometric fit and atomic desolvation energy. The 1000 best docked candidate transforms from PatchDock, based on global energy, aVdW, rVdW, atomic contact energy, and insideness measurements, were then used in FireDock (http://bioinfo3d.cs.tau.ac.il/) [34]. FireDock optimized, refined and rescored the 10 top candidate solutions by restricting the flexibility to the side-chains of the interacting surface and allowing small rigid-body movements. For this study, we selected the first best candidate solution from FireDock for the ApoE2-, ApoE3-, and ApoE4-A*β* complex.

### Molecular Dynamics simulation

Energy minimization, equilibration and molecular dynamics simulations were carried out at neutral pH using the GROMACS3.3.2 software with GROMOS96 as the force field [31]. The complexes of each of the three ApoE isoforms with A*β* peptide from the above-mentioned docking were used as the starting points for the simulations. Bond lengths were constrained using the LINCS algorithm and the SETTLE algorithm was used for hydrogen bonding of water. First, macromolecules from the docking model were solvated in a cubic box of 8Å cutoff with TIP3P water. Each complex was minimized with 2000 steps using the steepest descent algorithm in order to relieve bad interactions between ApoE and A*β* peptide. The system was equilibrated by first running 10 ps of position-restrained molecular dynamics; then the temperature of the system was gradually increased to 300 K. Berendsen's temperature coupling method (time constant of 0.1 ps) was used in an unrestrained simulation. Water molecules were equilibrated in the presence of the protein complex for 10 ps before running an unrestrained molecular dynamics simulation for 10 ns. For unrestrained molecular dynamics simulation, the temperature coupling and pressure coupling were conducted in the NpT ensemble by using a Berendsen thermostat of 300 K and 0.1 ps relaxation time. The pressure was 0.5 bar with 0.000045 compressibility and 1ps relaxation time, respectively. The simulations with 300 K were applied by 173529 seeds. Isotropic pressure coupling and Berendsen's temperature coupling were then used during a 10 ns molecular dynamics simulation. In addition, two MD simulations were run involving the three ApoE isoforms alone, following the above-mentioned process. All molecular representations in this study were generated using Chimera v1.4 (http://www.cgl.ucsf.edu/chimera/) [35]. The g_rms and g_dist of GROMACS3.3.2 were used to analyze the MD results.

## Supporting Information

Figure S1 FireDock Clustering. Clustering of the ten lowest energy solutions ranked by FireDock for ApoE2 (A, docking energies ranging from −64.52 to −45.22 Kcal/mol); ApoE3 (B, docking energies ranging from −62.70 to −48.30 Kcal/mol); and ApoE4 (C, docking energies ranging from −60.94 to −45.43 Kcal/mol). The lowest energy solution for A*β* is represented as a golden ribbon (ApoE is displayed as blue ribbons). The subsequent nine solutions for A*β* are plotted as grey cylinders.
(0.14 MB PDF)
