# Supplementary figures and images for: In Silico Analysis of the Apolipoprotein E and the Amyloid β Peptide Interaction: Misfolding Induced by Frustration of the Salt Bridge Network
Source: PLoS Comput Biol. 2010 Feb 5;6(2):e1000663. doi: 10.1371/journal.pcbi.1000663 (PMC2816681; doi:10.1371/journal.pcbi.1000663)

A

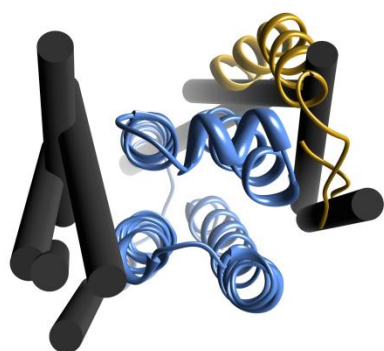

B

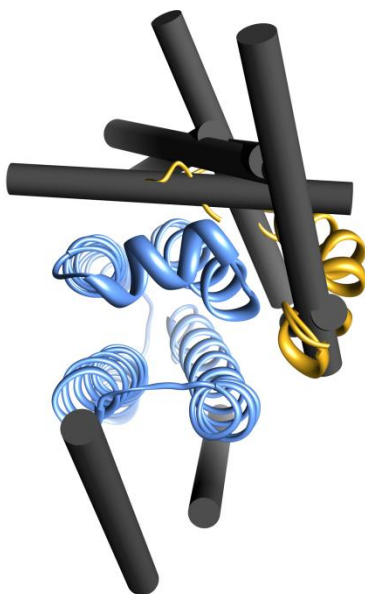

C

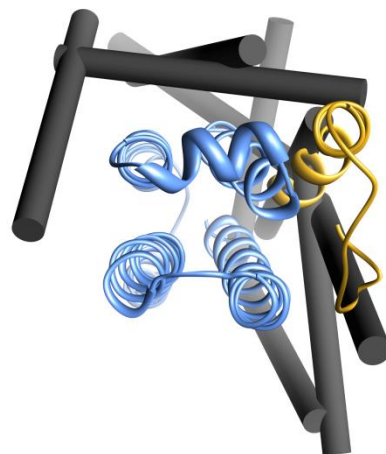

Supplement: Figure S1 — FireDock Clustering. Clustering of the ten lowest energy solutions ranked by FireDock for ApoE2 (A, docking energies ranging from −64.52 to −45.22 Kcal/mol); ApoE3 (B, docking energies ranging from −62.70 to −48.30 Kcal/mol); and ApoE4 (C, docking energies ranging from −60.94 to −45.43 Kcal/mol). The lowest energy solution for Aβ is represented as a golden ribbon (ApoE is displayed as blue ribbons). The subsequent nine solutions for Aβ are plotted as grey cylinders. (0.14 MB PDF) [file pcbi.1000663.s001.pdf]
